# Detection of MET Alterations Using Cell Free DNA and Circulating Tumor Cells from Cancer Patients

**DOI:** 10.3390/cells9020522

**Published:** 2020-02-24

**Authors:** Patricia Mondelo-Macía, Carmela Rodríguez-López, Laura Valiña, Santiago Aguín, Luis León-Mateos, Jorge García-González, Alicia Abalo, Oscar Rapado-González, Mercedes Suárez-Cunqueiro, Angel Díaz-Lagares, Teresa Curiel, Silvia Calabuig-Fariñas, Aitor Azkárate, Antònia Obrador-Hevia, Ihab Abdulkader, Laura Muinelo-Romay, Roberto Diaz-Peña, Rafael López-López

**Affiliations:** 1 Liquid Biopsy Analysis Unit, Translational Medical Oncology (Oncomet), Health Research Institute of Santiago (IDIS), 15706 Santiago de Compostela, Spain; Patricia.Mondelo.Macia@sergas.es (P.M.-M.); Alicia.Abalo.Pineiro@sergas.es (A.A.); oscar.rapado@rai.usc.es (O.R.-G.); rafael.lopez.Lopez@sergas.es (R.L.-L.); 2 Department of Medical Oncology, Complexo Hospitalario Universitario de Santiago de Compostela (SERGAS), 15706 Santiago de Compostela, Spain; rodriguezlopez.carmela@gmail.com (C.R.-L.); Santiago.Aguin.Posada@sergas.es (S.A.); Luis.Angel.Leon.Mateos@sergas.es (L.L.-M.); jorge.j.garcia.gonzalez@gmail.com (J.G.-G.); 3 Translational Medical Oncology (Oncomet), Health Research Institute of Santiago (IDIS), 15706 Santiago de Compostela, Spain; mariamercedes.suarez@usc.es (M.S.-C.); mtcuriel@yahoo.es (T.C.); 4 Department of Laboratory Medicine, Hospital Universitari Son Espases, 07120 Palma, Balearic Islands, Spain; valina@ssib.es; 5 Group of Advanced Therapies and Biomarkers in Clinical Oncology, Institut d’Investigació Sanitària de les Illes Balears (IdISBa), 07120 Palma, Balearic Islands, Spain; aitor.azkarate@ssib.es (A.A.); antonia.obrador@ssib.es (A.O.-H.); 6 Centro de Investigación Biomédica en Red de Cáncer (CIBERONC), 28029 Madrid, Spain; Angel.Diaz.Lagares@sergas.es (A.D.-L.); calabuix_sil@gva.es (S.C.-F.); 7 Department of Surgery and Medical Surgical Specialties, Medicine and Dentistry School, Universidade de Santiago de Compostela, 15705 Santiago de Compostela, Spain; 8 Cancer Epigenomics, Oncomet, Health Research Institute of Santiago (IDIS), Complexo Hospital Univeritario de Santiago de Compostela (SERGAS), 15706 Santiago de Compostela, Spain; 9 Molecular Oncology Laboratory, Fundación Hospital General Universitario de Valencia, 46014 Valencia, Spain; 10 Department of Pathology, Universitat de València, 46010 València, Spain; 11 TRIAL Mixed Unit, Centro de Investigación Príncipe Felipe-Fundación para la Investigación del Hospital General Universitario de València, 46012 València, Spain; 12 Medical Oncology Department, Hospital Universitari Son Espases, 07120 Palma, Balearic Islands, Spain; 13 Molecular Diagnosis Unit, Hospital Universitari Son Espases, 07120 Palma, Balearic Islands, Spain; 14 Department of Pathology, Complexo Hospital Universitario de Santiago de Compostela (SERGAS), Universidade de Santiago de Compostela, 15706 Santiago de Compostela, Spain; Ihab.Abdulkader.Nallib@sergas.es; 15 Faculty of Health Sciences, Universidad Autónoma de Chile, Talca 3460000, Chile

**Keywords:** MET protein expression, *MET* amplification, circulating free DNA (cfDNA), circulating tumor cells (CTCs), targeted therapy, *MET* copy number

## Abstract

MET alterations may provide a potential biomarker to evaluate patients who will benefit from treatment with MET inhibitors. Therefore, the purpose of the present study is to investigate the utility of a liquid biopsy-based strategy to assess MET alterations in cancer patients. We analyzed *MET* amplification in circulating free DNA (cfDNA) from 174 patients with cancer and 49 healthy controls and demonstrated the accuracy of the analysis to detect its alteration in patients. Importantly, a significant correlation between cfDNA concentration and *MET* copy number (CN) in cancer patients (*r* = 0.57, *p* <10^−10^) was determined. Furthermore, we evaluated two approaches to detect the presence of MET on circulating tumor cells (CTCs), using the CellSearch^®^ and Parsortix systems and monitored patients under anti-EGFR treatment (*n* = 30) combining both cfDNA and CTCs analyses. This follow-up provides evidence for the potential of *MET* CN assessment when patients develop resistance to anti-EGFR therapy and a significant association between the presence of CTCs MET^+^ and the Overall Survival (OS) in head and neck cancer patients (P = 0.05; HR = 6.66). In conclusion, we develop specific and noninvasive assays to monitor MET status in cfDNA/CTCs and demonstrate the utility of plasma *MET* CN determination as a biomarker for monitoring the appearance of resistance to anti-EGFR therapy.

## 1. Introduction

Receptor tyrosine kinases (RTKs) are crucial regulators of key cellular processes such as cell growth, differentiation, neovascularization, and tissue repair. Hepatocyte growth factor receptor (MET or c-MET) is an RTK produced predominantly in cells of epithelial origin [1]. Its only known high-affinity ligand is the hepatocyte growth factor (HGF) [2] and both are essential to embryonic development and organ regeneration. The binding of MET and HGF activates the kinase activity of MET and several pathways, such as the mitogen-activated protein kinase (MAPK) cascade, the dylinositol 3-kinase pathway (PIK3K-Akt), the signal transducer and activator of transcription (STAT) pathway, and the IκBα–NF-κB complex [3]. These pathways can activate cell proliferation, survival, migration, motility, invasion, angiogenesis, apoptosis, and epithelial-to-mesenchymal transition [3,4]. In addition, MET can interact with other cell membrane receptors, such as integrins, CD44, class B Plexins, and other tyrosine kinase receptors (e.g., HER2, AXL, EGFR, and VEGF) [1,2].

Deregulation of the MET pathway has been associated with cancer growth and metastasis in several types of tumors (lung, head and neck, gastric, and colorectal, among others) [1,3], taking place through several mechanisms including overexpression, amplification, autocrine signaling, and mutational activation [5]. In addition to its role as an oncogenic driver, MET alterations have been described as a mechanism of resistance to different therapies [1,6,7]. Therefore, several clinical trials have assessed the efficacy of selective and broad-spectrum MET inhibitors in an extensive panel of cancers [8], generating interest in MET activity as a promising target for anticancer therapy.

MET protein overexpression and/or amplification are frequently found in different cancers. In fact, *MET* amplification has been also described in diverse tumor types, such as non-small cell lung cancer (NSCLC), gastric cancer, esophageal cancer, colorectal cancer, medulloblastoma, and glioblastoma, and has been associated with bad prognosis and poor survival [1,9,10,11,12]. Furthermore, this alteration is more frequent in metastatic patients and has been specifically associated with the development of resistance to anti-EGFR therapy [1,9]. On the other hand, MET overexpression is known to activate the MET-signaling pathway, promoting tumor cell growth, survival, migration, and invasion. This alteration has been associated with bad prognosis and the generation of metastasis in different tumor types, such as NSCLC, hepatocellular carcinoma, kidney cancer, head and neck cancer, colorectal cancer, gastric cancer, nasopharyngeal carcinoma, and glioblastomas [2,3,6,11,12,13,14]. In addition, MET overexpression has been associated with chemotherapy and radiotherapy resistance in breast cancer [1,6,7].

All these data indicate that *MET* amplification and/or MET overexpression may be a potential biomarker for the evaluation of patients who will benefit from treatment with MET inhibitors [14]. However, there are no standardized methods at present to confirm these molecular alterations. In fact, variable *MET* amplification rates can be detected, depending on the detection techniques [14], for example, fluorescence in situ hybridization (FISH), single-nucleotide polymorphism (SNP) genotyping, or quantitative polymerase chain reaction (qPCR), in which different scoring criteria may define high amplification.

In the same line, MET protein levels can be dependent on the antibodies employed, which can recognize different MET epitopes and domains, showing different membrane and/or cytoplasmic staining intensities by immunohistochemistry (IHC) [14]. Moreover, *MET* genomic changes have mostly been associated with metastatic patients and normally appear with the progression of disease [5]. Therefore, tissue re-biopsy constitutes the best alternative for such molecular analysis in tissue samples; however, re-biopsy is often not possible, making the validation of liquid biopsy strategies to address MET status essential. In fact, circulating tumor cells (CTCs) and circulating free DNA (cfDNA) represent an accessible and noninvasive alternative for detecting MET alterations in patient’s blood, particularly in patients for whom tissue biopsies are inaccessible or problematic to carry out or repeat [15]. Therefore, the purpose of the present study was to investigate the utility of a liquid biopsy-based strategy to assess MET alterations in cancer patients. For this aim, we evaluated the *MET* copy number (CN) status in cfDNA and the MET expression in CTCs from a cohort of cancer patients with different tumor types and evaluated its clinical potential to detect the appearance of resistances and to guide the treatment with anti-MET drugs in a noninvasive manner.

## 2. Materials and Methods

### 2.1. Cell Lines

The MET Genetic Alteration Cell Panel was purchased from the American Type Culture Collection (ATCC, TCP-1036). The panel was composed of five human tumor cell lines (SNU-5, Hs746T, C32, AU565, and NCI-N87) showing various degrees of *MET* gene CN changes and expression levels. We also included the MET-negative prostate cancer cell line LNCaP and the cancer cell lines A549 (lung, NSCLC) and PC3 (prostate), purchased from the ATCC, in the study. The cell lines were routinely cultured in ATCC’s recommended growth medium at 37 °C, 5% CO_2_, and 95% humidity.

*MET* CN data of the eight cell lines were obtained from the Cancer Cell Line Encyclopedia (CCLE) database (http://www.broadinstitute.org/ccle/home), determined using the Affymetrix Genome-Wide Human SNP Array 6.0 platform. Next, genomic DNA (gDNA) was extracted from the same cancer cell lines with a QIAamp DNA Blood Mini Kit (Qiagen, Hilden, Germany), according to the manufacturer’s suggested protocol. DNA quantity was assessed using a NanoDrop spectrophotometer (Thermo Fisher Scientific, Waltham, MA, USA).

### 2.2. Patients and Samples

The study design for testing the utility of blood samples to assess the MET status in cancer patients is shown in Supplementary Figure S1. We designed a prospective study to analyze *MET* amplification, including 112 patients diagnosed with diverse metastatic cancers (such as NSCLC, colorectal cancer, head and neck, or melanoma, among others) who had progressed to at least one line of therapy and also 34 patients with non-metastatic tumors. They were recruited between August 2017 and October 2019 at the Medical Oncology Service of Complexo Hospitalario Universitario de Santiago de Compostela and Hospital HM La Rosaleda of Santiago de Compostela. We also included 28 stage IV NSCLC EGFR mutated patients treated with tyrosine kinase inhibitors at the Medical Oncology Service of Hospital Universitario Son Espases (Mallorca, Spain) (Table 1, Supplementary Table S1). Frozen plasma samples from this last cohort, collected between September 2016 and November 2019, were retrospectively assessed for *MET* CN. These patients had a progression to first-line tyrosine kinase inhibitors (TKIs) and some of them even to osimertinib in second-line (irreversible EGFR TKI). In addition, we recruited 49 healthy volunteers as controls for testing the specificity of the *MET* CN analyses. Among the patients recruited, those diagnosed with lung or head and neck cancers starting with anti-EGFR treatment (*n* = 30), were monitored by means of cfDNA and CTCs analysis before the initiation of therapy (baseline), at 12 and 24 weeks after the onset of therapy, and at disease progression in order to evaluate the dynamics of *MET* amplification and overexpression within tumor evolution.

All individuals gave written informed consent approved by Santiago de Compostela and Lugo, and Islas Baleares Ethics Committees (Ref: 2017/538 and IB2584/15; respectively) prior to enrolling in the study. The protocol approved by the Ethics Committee was conducted according to the Declaration of Helsinki.

### 2.3. Circulating Free DNA Isolation from Plasma Samples

Ten milliliters of peripheral whole blood from controls and patients were obtained by direct venipuncture directly with Streck Cell-Free DNA blood collection tubes (Streck, La Vista, NE, USA). Plasma was separated within 96 h after blood collection as a result of two sequential centrifugation steps (10 min at 1600 *g*, and 10 min at 6000 *g*; both at room temperature), and then stored at -80 °C for further processing. cfDNA was extracted from 3 mL of plasma using a QIAmp Circulating Nucleic Acid Kit (Qiagen, Hilden, Germany), according to the manufacturer’s instructions. cfDNA yields were assessed using a Qubit 1X dsDNA Hs Assay Kit, according to the manufacturer’s instructions (Thermo Fisher Scientific, USA).

### 2.4. Droplet Digital PCR to Detect MET CN

Genomic DNA extracted from the cancer cell lines (*n* = 8) and cfDNA isolated from plasma (*n* = 140 metastatic patients, *n* = 34 non-metastatic patients, and *n* = 49 healthy controls) was amplified using ddPCR to analyze *MET* CN. The ddPCR experiments were performed according to the manufacturer’s protocol (Bio-Rad, Hercules, CA, USA). Each assay contained 20 μL reaction mixture of 10 μL ddPCR Supermix for Probes (Bio-Rad, CA, USA), 1 μL PrimePCR ddPCR CN assay for *MET* with a FAM fluorophore (dHsaCP2500321, Bio-Rad, CA, USA), 1 μL PrimePCR ddPCR CN assay for the reference gene with a HEX fluorophore (*EIF2C1*, dHsaCPE2500349, Bio-Rad, CA, USA), and 8 μL DNA template and nuclease-free water. In the case of gDNA from cancer cell lines, we added the HaeIII enzyme (Takara Bio, USA) to the mix, according to the manufacturer’s instructions. Droplets were generated using a QX200 AutoDG Droplet Digital PCR System (Bio-Rad, CA, USA) with Droplet Generation Oil for Probes (Bio-Rad, CA, USA) and transferred to a 96-well plate. PCR amplification was performed with the following conditions: 95 °C for 10 min, followed by 40 cycles of 94 °C for 30 s, 60 °C for 60 s, and 10 min incubation at 98 °C. The plates were read on a Bio-Rad QX200 droplet reader (Bio-Rad, CA, USA) and we calculated *MET* CN using the QuantaSoft Pro software (Bio-Rad, CA, USA).

### 2.5. Fluorescent In Situ Hybridization (FISH) Analysis

Fluorescent In Situ Hybridization (FISH) analysis was conducted on formalin-fixed paraffin-embedded (FFPE) tissues in a subgroup of patients who had a blood sample taken within 2 months after tissue biopsy (*n* = 12). The tissue sections were hybridized with MET (SpectrumRed)/CEN7 (SpectrumGreen) Dual Color FISH Probe (Vysis, Abbott Molecular) following the manufacturer’s protocol. In each case, 60 non-overlapping nuclei were examined by a fluorescence microscope (BX51, Olympus Corporation, Barcelona, Spain). FISH positivity was estimated using the standard Colorado criteria (gene amplification; 2 > *MET* gene (red)/CEP7q (green) per cell plus high polysomy; 5 copies > mean *MET* per cell) [16].

### 2.6. Spiked Experiments

The assays to evaluate MET expression on CTCs were tested using cancer cell lines spiked in whole blood from the healthy volunteers recruited. A total of 7.5 mL of blood from healthy donors was collected in CellSave tubes (Menarini, Silicon Biosystems, Bologna, Italy). Cells were spiked at a final concentration of 30 cells per mL of blood. Concurrently, we employed samples from three healthy donors per each cell line. These samples were analyzed using the CellSearch^®^ and Parsortix Systems, running identical blood samples with both technologies. All spiked samples were enriched within 96 h of collection.

### 2.7. Analysis of MET Expression on CTCs Isolated Using Cell Search^®^

A total of 7.5 mL of peripheral whole blood samples were collected in CellSave tubes (Menarini, Silicon Biosystems, Bologna, Italy) for CTCs enumeration using the FDA-cleared CellSearch^®^ system (Menarini, Silicon Biosystems, Bologna, Italy). CellSearch^®^ Circulating Tumor Cell Kit (Menarini, Silicon Biosystems, Bologna, Italy) was used for these specific experiments, including ferrofluids coated with epithelial cell-specific anti-Epithelial cell adhesion molecule (EpCAM) antibodies to immunomagnetically enrich epithelial cells; a mixture of antibodies directed to cytokeratins (CKs) 8, 18, and 19 conjugated to phycoerythrin (PE); anti-CD45 mAb conjugated to allophycocyanin (APC); and nuclear dye 4′,6-diamidino-2-phenylindole (DAPI) to fluorescently label the cells. The C32 cells and human melanoma samples were analyzed using a CellSearch^®^ Circulating Melanoma Cell Kit (Menarini, Silicon Biosystems, Bologna, Italy), which included ferrofluid coated with antibodies targeting the CD146 antigen to capture circulating melanoma cells (CMCs) and fluorescent reagents against anti-high molecular weight melanoma-associated antigen (HMW-MAA-PE (MEL-PE)), anti-CD34-APC for endothelial cells, anti-CD45-APC for leukocytes, and DAPI for staining cell nuclei. The open 4th antibody position of the CellSearch^®^ system was used to evaluate MET expression. We employed the anti-MET fluorescein (FITC)-conjugated monoclonal antibody (D-4, sc-514148, Santa Cruz, CA, USA) at a final concentration of 20 μg/mL. CTCs were identified as EpCAM^+^, CK^+^, CD45^−^, and DAPI^+^, and the level of MET expression was recorded for each CTC, on a 0–3 scale, by comparison to the MET expression levels of the different cell lines reference.

### 2.8. Analysis of MET Expression on CTCs Isolated Using Parsortix System

A total of 7.5 mL of peripheral whole blood was collected in CellSave tubes (Menarini, Silicon Biosystems, Bologna, Italy) and loaded into the Parsortix microfluidic device (Angle Inc, Guildford, UK). CTCs were then enriched from blood samples in disposable Parsortix cassettes (GEN3D6.5, Angle Inc, Guildford, UK), according to the manufacturer’s guidelines. CTCs were trapped in the Parsortix cassette due to their large size and lower compressibility than the remaining blood cells. After separation, we fixed the sample and carried out immunofluorescence staining. CTCs were subjected to on-cassette-staining with selected antibodies, according to the manufacturer’s guidelines, followed by fluorescence microscopy detection (Leica DMI8, Leica Microsystems, Germany). The selected antibodies included Alexa Fluor (AF)546-conjugated pan-Cytokeratin (C11, sc-8018, Santa Cruz, CA, USA), AF647-conjugated CD45 (35-Z6, sc-1178, Santa Cruz, CA, USA), anti-MET FITC-conjugated (D-4, sc-514148, Santa Cruz, CA, USA), and DAPI to fluorescently label the cells. CTCs were identified as CK^+^, CD45^−^, and DAPI^+^, and the level of MET expression on CTCs was determined, on a scale of 0–3 scale, depending on the MET antibody intensity, according to the results obtained in cell-line controls.

### 2.9. Statistical Analysis

Statistical analyses were performed using R version 3.4.0. Pearson’s correlation and linear regression were used to evaluate the relationship between ddPCR and SNP6.0 or FISH results. The Mann–Whitney–Wilcoxon U-Test was used to compare the *MET* CN between cancer patients and healthy controls. Two-tailed P values of 0.05 or less were considered significant. The cut-off value for the *MET* CN test to define the presence of *MET* amplification was determined using the mean plus 3 standard deviations of healthy controls. The Kappa test was used to determine the concordance between *MET* amplification based on FISH and ddPCR analyses. Paired Student t tests were used to compare the CTCs enrichment potential between the Parsortix and the CellSearch^®^ systems.

## 3. Results

### 3.1. Accuracy of ddPCR to Detect MET CN Alterations

**Figure 1:**
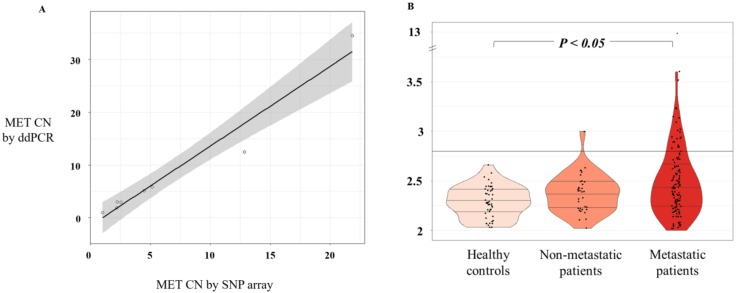
*MET* CN analysis. (**A**) Scatterplot representing correlation between *MET* CN in cancer cell lines determined by ddPCR versus single-nucleotide polymorphism (SNP) array (*n* = 8) using Pearson’s correlation; (**B**) Plasma *MET* CN detected in healthy controls (*n* = 49), non-metastatic patients (*n* = 34), and metastatic patients (*n* = 140) using the Mann–Whitney–Wilcoxon U-Test.

We optimized the ddPCR assay using DNA isolated from a panel of cell lines representative of different levels of *MET* amplification and different cancer tumor types (lung, colon, prostate, skin, breast, and gastric). First, we obtained *MET* CN data as determined by microarray (Affimetrix SNP6.0 Array) from the publicly available Cancer Cell Line Encyclopedia database. Then, we compared the data with our ddPCR results in the same cancer cell lines. After the comparative analysis, a strong linear association of *MET* CN measurements based on Pearson’s correlation (r = 0.98; *p* < 10^-4^) was observed, validating the accuracy of our analytic approach (Figure 1A). We also used serial dilutions of cfDNA samples and genomic DNA from the cancer cell lines representing the different CN values (Supplementary Figure S2). We concluded that the minimum amount of DNA to detect *MET* CN was 2.3 ng total in each ddPCR reaction.

### 3.2. MET CN Assessment in cfDNA from Cancer Patients and Healthy Controls

We evaluated *MET* CN in cfDNA from the plasma of 140 patients with metastatic cancer progressing to at least one treatment, 34 non-metastatic patients, and 49 healthy controls. The control group was employed to set the normal values associated with a non-amplified *MET* status. Therefore, based on the CN values in the control population (*MET* CN between 1.9 and 2.66), a threshold of 2.8 was established to define the presence of *MET* amplification (Figure 1B). Importantly, CN values in patients were found between 1.75 and 12.7 (Figure 1B) and the comparison of the *MET* CN values between metastatic patients and healthy controls showed a statistically significant difference between the two populations (*p* < 0.05; Mann–Whitney test; Figure 1), reinforcing the accuracy of our analytical strategy. In fact, 23 values from the metastatic patients were higher than 2.8, being considered as positive for *MET* amplification. The CN range in the non-metastatic cohort was similar to the healthy controls, and only one patient showed a *MET* amplification.

**Figure 2:**
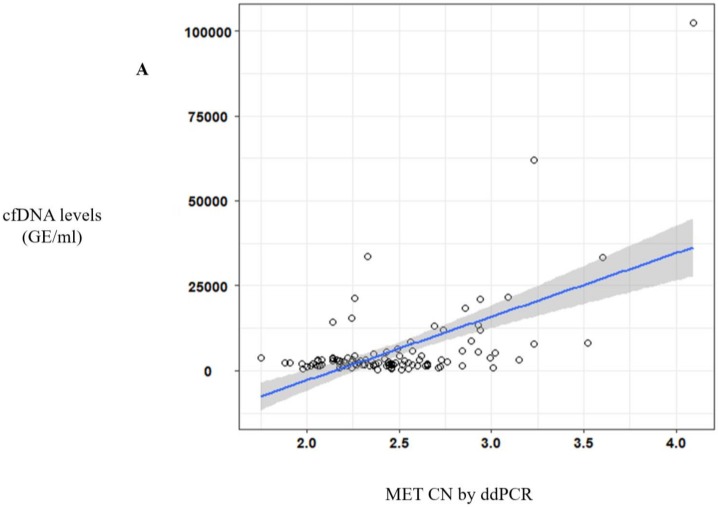

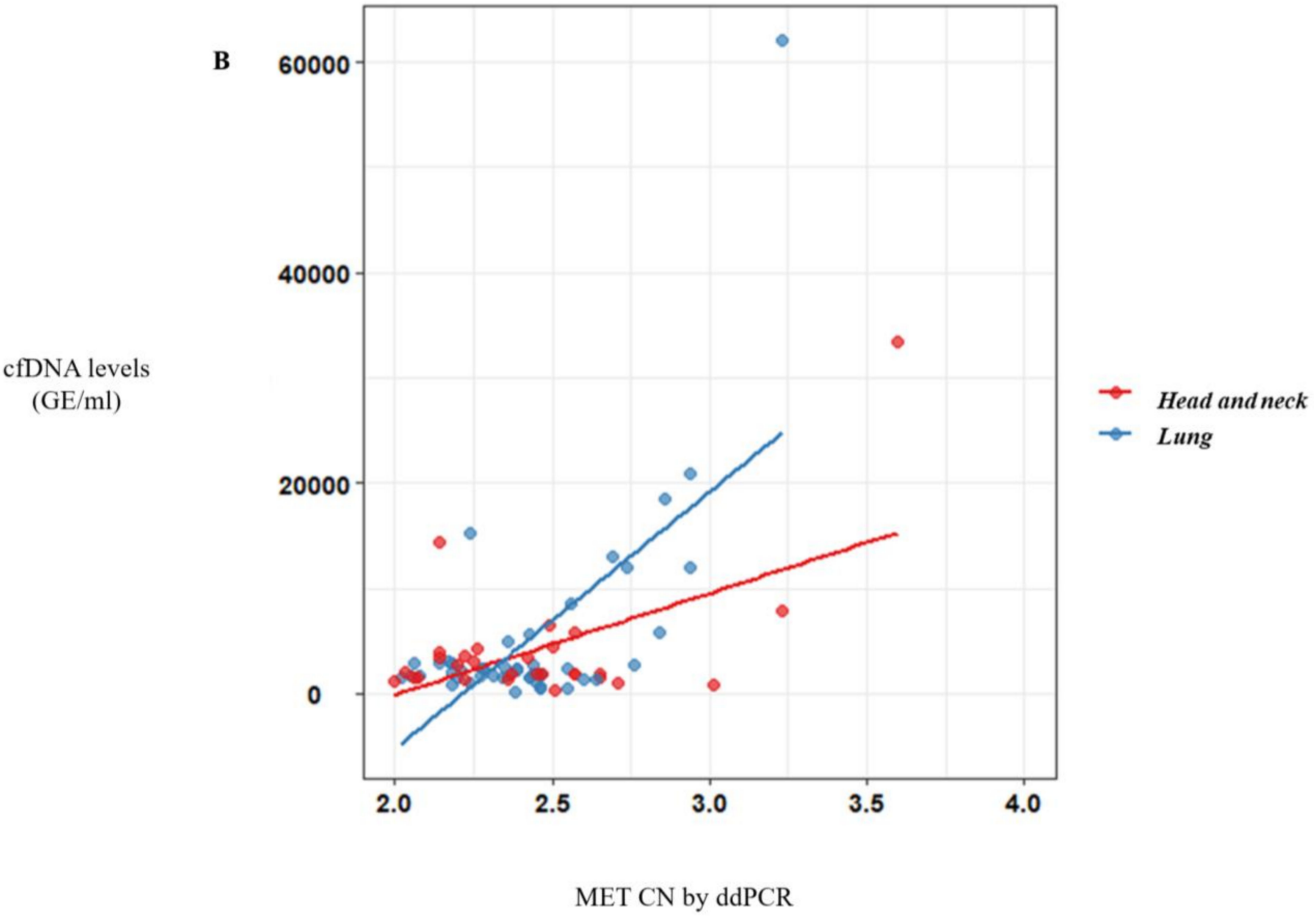
*MET* CN analysis in circulating free DNA (cfDNA) from metastatic cancer patients. (**A**) Correlation between cfDNA levels and plasma *MET* CN in all metastatic cancer patients (*n* = 140); (**B**) Correlation between cfDNA levels and plasma *MET* CN in lung and head and neck cancer patients (*n* = 30).

Of note, a significative correlation between cfDNA concentration and *MET* CN was observed (*r* = 0.57, *p* < 10^−10^) (Figure 2A) in metastatic patients evidencing the higher capacity to detect *MET* amplification in patients with high cfDNA levels. Besides, when we analyzed the most represented types of cancer—NSCLC and head and neck—this positive correlation was still observed (*r* = 0.69, *p* < 10^−6^ and *r* = 0.56, *p* < 0.005, respectively; Figure 2B).

On the other side, no association was found between *MET* CN values and clinical characteristics, such as the number of metastatic sites or the treatment lines received prior to sample collection. We also found a high variability of the *MET* CN status between the different tumor types, which was not significantly different among groups. However, it is remarkable that all patients with renal cancer (*n* = 3) showed *MET* CN values over the threshold (2.89, 2.92, and 3.09 values) (Table 1; Supplementary Figure S3).

### 3.3. MET Amplification Concordance between cfDNA and Tissue Samples

**Figure 3:**
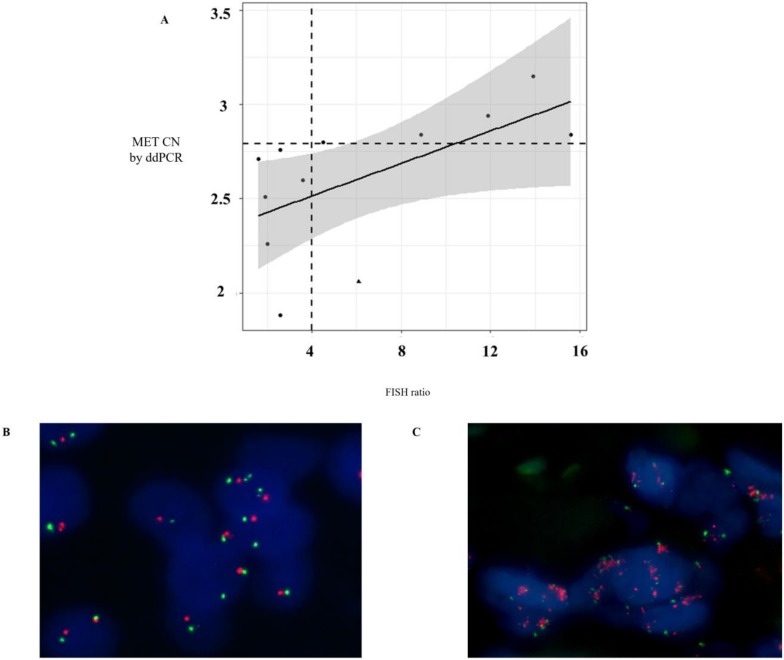
Comparison of *MET* CN status in tissue and cfDNA. (**A**) Distribution of *MET* CN measured by ddPCR and fluorescence in situ hybridization (FISH) (the point larger indicates the discordant value, whereas the horizontal and vertical dotted lines indicate cut-off points of ddPCR and FISH, respectively); (**B**) Representative example of a negative case for *MET* amplification obtained in a NSCLC patient by FISH; and (**C**) Representative example of a positive case for *MET* amplification obtained in a NSCLC patient by FISH.

Currently, in routine studies, FISH is the standard method used to evaluate the *MET* CN [16,17]. We tested the concordance of *MET* amplification between cfDNA and tissue samples in 12 patients with melanoma (*n* = 4), lung (*n* = 3), ovarian (*n* = 2), head and neck (*n* = 2), and skin cancers (*n* = 1) by ddPCR and FISH, respectively, in order to evaluate the sensitivity and specificity of our *MET* CN assay compared to the FISH ratio MET/CEN-7 > 4 (based on standard Colorado criteria). It is important to remark that, in these patients, the date of tissue and plasma sampling collection was less than 2 months. The linear association between *MET* CN analyzed by ddPCR and the MET/CEN-7 ratio analyzed by FISH was relatively moderate (*r* = 0.58; *p* < 0.05; Figure 3A), while the concordance rate was 91.67% with Cohen’s kappa of 0.833 (95% CI: 0.615–0.998, *p*-value < 0.005). Sensitivity and specificity to detect the *MET* amplification present in tissue by means of plasma ddPCR were 85.71% and 100%, respectively, demonstrating a good performance of the technique.

### 3.4. MET Expression Analysis on CTCs

To complement our approach in characterizing MET status by means of liquid biopsy, we developed a strategy to assess MET expression in the CTCs population. First, we evaluated the enrichment potential of the CellSearch^®^ and Parsortix systems, using preserved blood samples from healthy controls spiked with six human tumor cells (LNCaP, NCI-N87, Hs746T, AU565, SNU-5, and C32), representative of the variability of *MET* CN and MET expression. The average percentage of spiked cells captured by the Parsortix system was 73.04 ± 19.23% (range 54–99.01%), while, for the CellSearch^®^ system, the average recovery was 71.60 ± 22.58% (range 32.65–91.32%).

**Figure 4:**
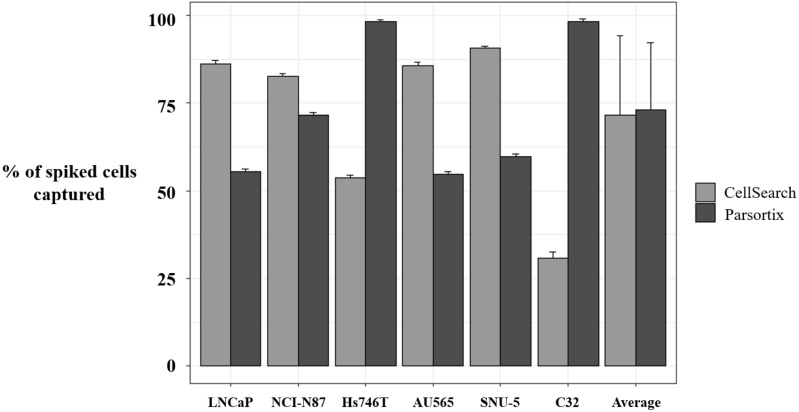
Percentage of spiked tumor cancer cells captured using CellSearch^®^ and Parsortix systems. Evaluation of the enrichment capacity of CellSearch^®^ and Parsortix systems, using healthy blood spiked with LNCaP, NCI-N87, Hs746T, AU565, SNU-5, and C32 cancer cell lines. LNCaP, NCI-N87, SNU-5, and AU565 express Epithelial cell adhesion molecule (EpCAM) while Hs746T and C32 express low levels or do not express EpCAM, respectively. *p*-value < 5 × 10^−3^, in all comparisons between CellSearch^®^ and Parsortix System in each cell line.

**Figure 5:**
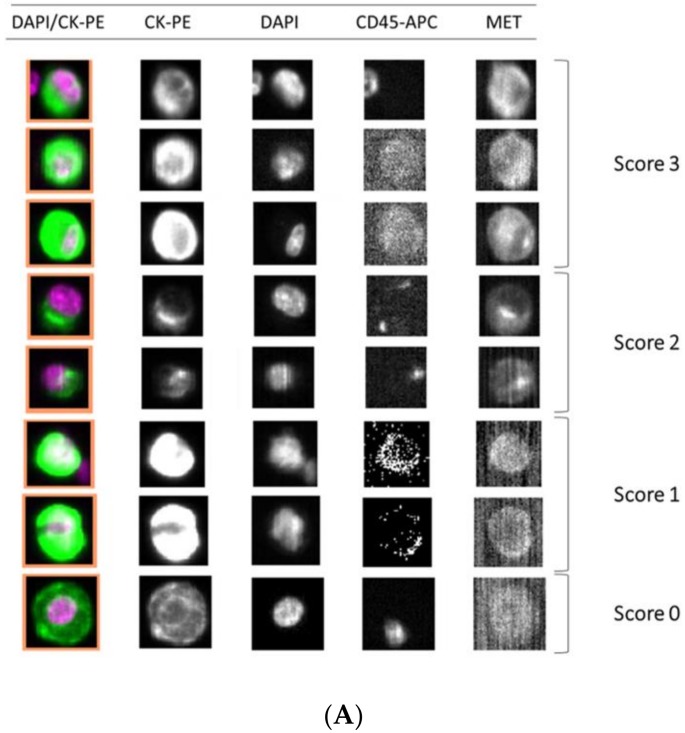

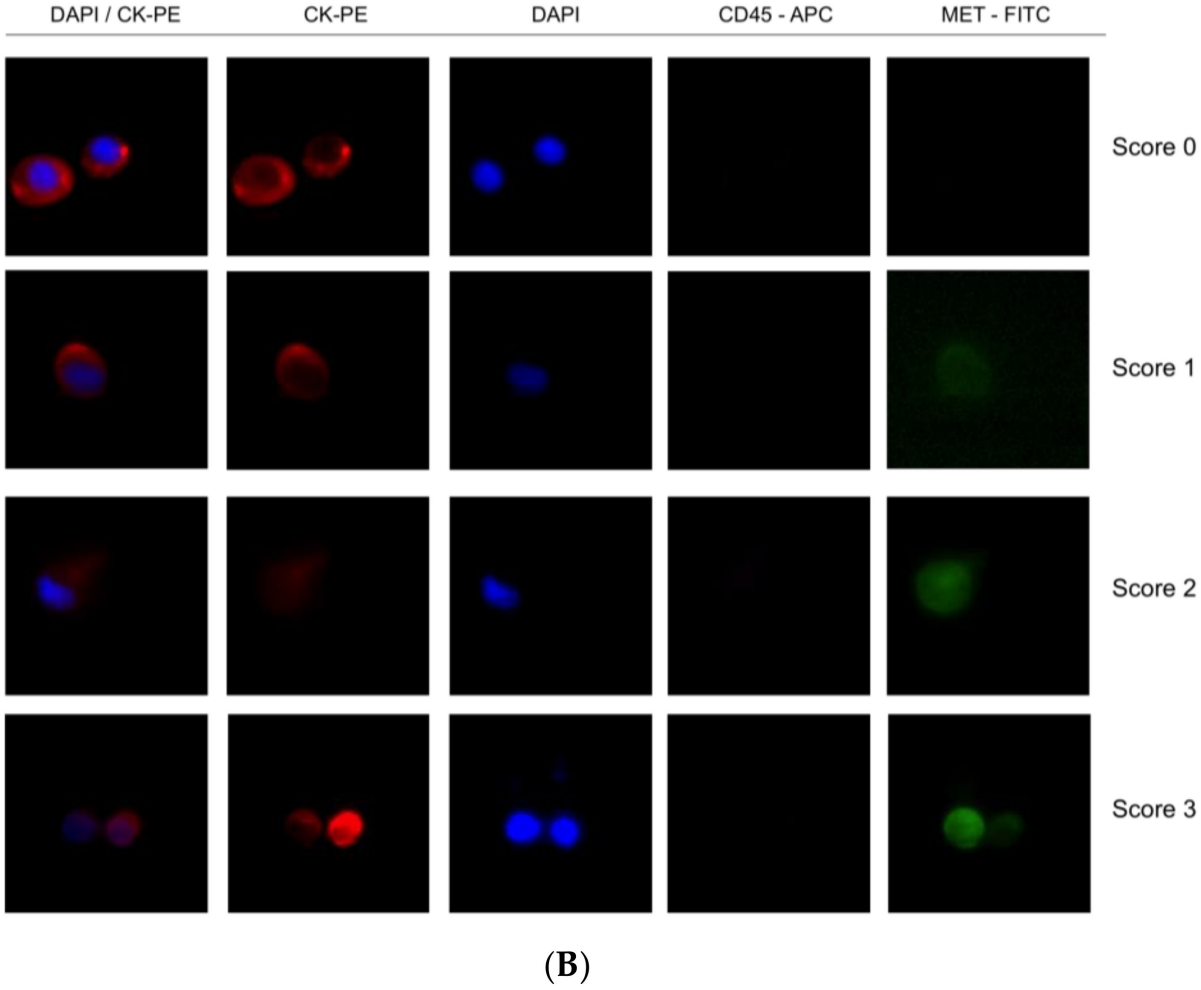
Detection of MET expression using tumor cancer cells with the CellSearch^®^ and Parsortix systems (**A** and **B**, respectively). Representative images of MET expression scored on score 0 (cell line LNCaP), 1 (cell line AU565), 2 (cell line Hs746T), and 3 (cell line SNU-5).

Importantly, we observed a different isolation efficacy with CellSearch^®^ and Parsortix platforms to isolate the cell lines as result of the differential strategy for the CTCs enrichment applied by both platforms (*p*-value < 5 × 10^−3^, in all comparisons; Figure 4). The Parsortix system that isolates CTCs based on their size and deformability showed higher isolation efficiency compared to the EpCAM-dependent enrichment method of CellSearch^®^ in the case of Hs746T and C32 cancer cell lines (*p*-value = 2.63 × 10^−5^ and 2.90 × 10^−7^, respectively). These cell lines are characterized by a low EpCAM expression explaining the low recovery with an EpCAM-dependent strategy (Supplementary Figures S4 and S5). Once the performance of both technologies was assessed, we established immunoscores for MET expression on single CTCs for both systems to grade this expression (Figure 5). We defined four different levels for the intensity of MET presence: score 0 (negative), 1+ (weak), 2+ (moderate), or 3+ (strong) based on the signal obtained in cells with 0 (LNCaP), 1+ (AU565), 2+ (Hs746T), or 3+ (SNU-5).

### 3.5. MET Amplification and CTCs Expression in Patients under Anti-EGFR Treatment

**Figure 6:**
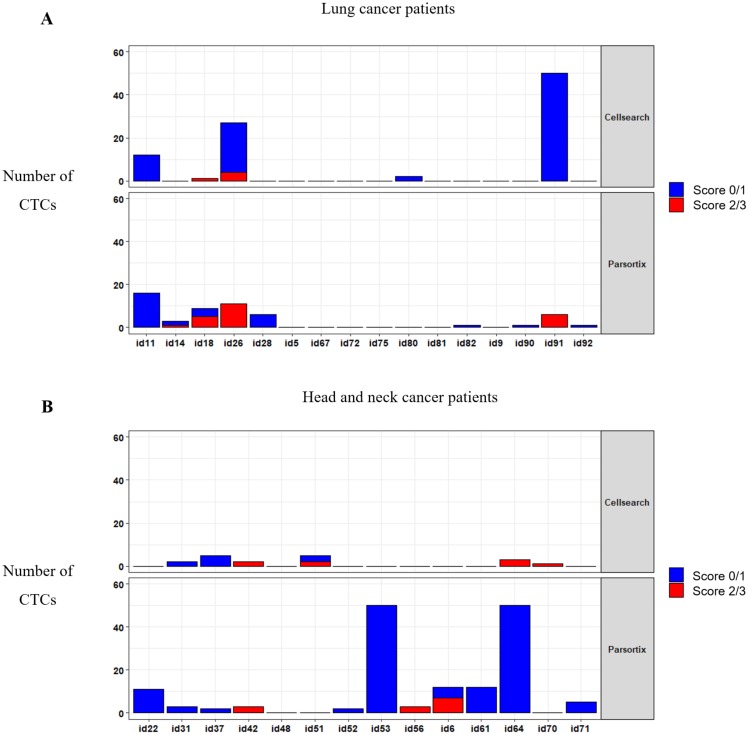
CTCs enumeration and MET expression in blood samples evaluated by the CellSearch^®^ (upper panel) and Parsortix (down panel) systems. Distribution of MET scores in CTCs from patients with NSCLC (**A**) and head and neck cancer (**B**).

To determine whether *MET* CN and expression can be used to monitor a patient´s response to anti-EGFR therapy, we applied both analyses in 30 patients with metastatic head and neck, or NSCLC at longitudinal sampling points (before the anti-EGFR therapy, at 12 and 24 weeks after the therapy onset, and at disease progression). We analyzed the MET expression in CTCs using the optimized conditions described above (Figure 6; Supplementary Table S2). At baseline, using the CellSearch^®^ system, at least one CTC was detected in 11 patients (36.67%), ranging from 1 to 77 CTCs in 7.5 mL of blood (mean = 4.83 CTCs). From these 11 patients, 6 showed MET overexpression (score 2 or 3), representing 20% of all patients analyzed. Using the Parsortix system, at least one CTC was detected in 20 patients (66.67%) at baseline, ranging from 1 to 83 CTCs in 7.5 mL of blood (mean = 8.8 CTCs), being MET overexpression found in 8 patients out of 30 (26.67%). These results evidence a higher efficiency of Parsortix system in comparison to CellSearch^®^, at baseline, in this cohort of patients with lung or head and neck tumors. In fact, 12 patients (7 patients with head and neck tumors and 5 with NSCLC) were positive for CTCs using Parsortix system but negative using CellSearch^®^ while the inverse situation was observed only in 3 patients. Therefore, the overall positive concordance of both strategies was 50%. Regarding MET status, in 4 patients, MET overexpression was found exclusively using Parsortix system, while 3 patients showed overexpression only using CellSearch^®^. Overall, 11 patients showed CTCs overexpressing MET using CellSearch^®^ or Parsortix system.

**Figure 7:**
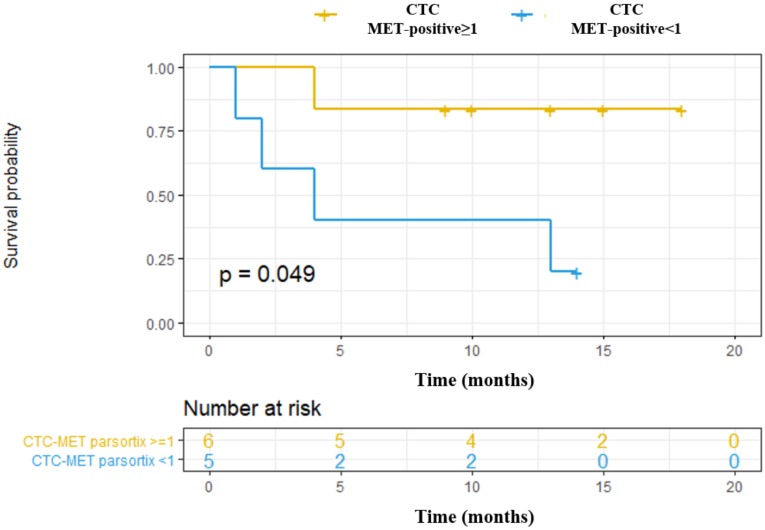
Prognostic value to predict Overall Survival (OS) of CTCs enumeration and MET expression in head and neck cancer patients starting with anti-EGFR treatment. CTCs MET-positive ≥1: CTCs with high MET expression (scores 2+ or 3+); CTCs MET-positive <1: CTC with low MET expression (score 1+).

We also explored the association of *MET* CN and MET overexpression at baseline with the Progression-Free Survival (PFS) and Overall Survival (OS) of these patients, as a “pilot” study. Using the Parsortix system, we found a significant association between CTCs MET-positive presence (CTC-MET-positive ≥1) and a poorer OS in head and neck cancer patients (*p*-value = 0.049, hazard ratio (HR) = 6.66; Figure 7). Although these results are of clinical interest, taking into account the small number of patients, and the even more, the low number of patients with MET expression in CTCs, they should be considered as preliminary. Of note, there was no relationship between *MET* amplification or MET expression in CTCs isolated by CellSearch^®^ and the patients’ survival, probably because of the limited population analyzed.

Longitudinal *MET* amplification and MET overexpression analyses were further addressed in 30 patients undergoing EFGR therapy. Eight of the 30 patients died and nine showed progressive disease during the follow-up. Five patients died before the second sampling point and 3 before the third collection point. Regarding CTCs monitoring using CellSearch^®^ system 4 of 12 patients were positive for CTCs and MET overexpression after 12 weeks of treatment, 3 of 9 after 24 weeks, and 1 of 8 at progression. In the case of Parsortix samples, 3 of 7 patients had CTCs with MET overexpression after 12 weeks, 0 of 5 after 24 weeks, and 2 of 9 at progression. In addition, 1 of 20 patients showed *MET* amplification after 12 weeks, 2 of 17 after 24 weeks, and 1 of 9 at progression, evidencing the amplification acquisition as a response of the treatment pressure. However, *MET* amplification and MET overexpression results only showed clear changes in a few of the patients within the treatment, suggesting its interest for monitoring, but these results should be considered as with caution because the longitudinal cohort was quite sparse.

**Figure 8:**
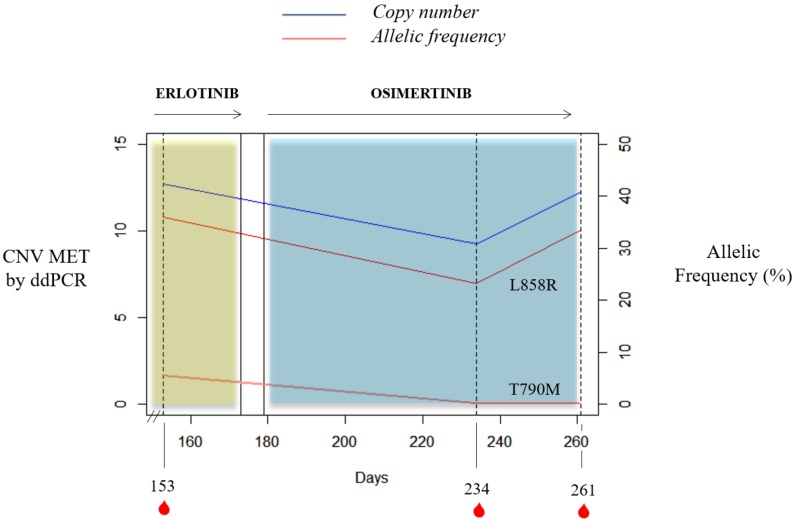
Timeline for the clinical course of patient id60. The blue and yellow bars represent the treatments time frame, and the red drops indicate blood collection time points. Percent mutant allelic frequency (L858R and T790M) and *MET* CN for patient id60 are shown.

It is important to mention the case of a stage IV NSCLC adenocarcinoma L858R carrier with bone lesions compatible with bone metastases. She was recruited at the start of erlotinib therapy and *MET* CN status in cfDNA and the MET expression in CTCs were also evaluated. She had 31 CTCs in the CellSearch^®^ system, 4 of them showing MET overexpression (12.9%); whereas 13 CTCs were detected with Parsortix system (11 MET-positive, 84.62%). Despite not showing radiological progression, there was a serious clinical deterioration and the patient died within two months. Besides, one case of the retrospective study evidenced how *MET* CN detection may be informative in monitoring the appearance of resistance mechanisms in response to anti-EGFR treatment (Figure 8). Longitudinal plasma samples from this 84-year-old female, diagnosed with stage IV NSCLC adenocarcinoma (patient id60) with metastases in bone and liver, were collected before osimertinib therapy (day 153), two months after the start of osimertinib therapy (day 234), and at disease progression (day 261). An EGFR mutation (L858R) was detected at the disease diagnosis and erlotinib, a first-generation EGFR tyrosine kinase inhibitor (TKI), was started. She progressed at day 145 (T790M-positive) and, 8 days after, the first plasma sample was collected. Next, the patient received osimertinib, a third-generation TKI, until progression (day 265) and she died on day 281.

## 4. Discussion

This study was designed to investigate the utility of liquid biopsy in assessing MET protein expression and *MET* CN, defined as MET status, in cancer patients. We used ddPCR to quantify *MET* CN in cfDNA and in a complementary way, we evaluated two approaches to detect MET presence on CTCs to overcome the complexity of isolating different phenotypes of CTCs (CellSearch^®^ and Parsortix systems).

For CTCs analysis, we implemented the MET expression detection using the CellSearch^®^ and the Parsortix platforms routine. Due to the ultra-rare nature of CTCs, with numbers as low as 1 CTC per 10^6^-10^7^ leukocytes, most assays for CTCs analyses use a combination of enrichment and detection/characterization techniques [18]. It is important to remark that the most widely used CTCs enrichment technique is the immunomagnetic-based selection of CTCs, being CellSearch^®^ system the only FDA approved device for CTCs enumeration, although there are others based on this strategy (e.g., IsoFlux™, AdnaTest, and MACS^®^). The main limitation of this approach is the lack of a “universal marker” that can be used independently of both the tumor type and the stage of disease progression [19]. To solve this problem, new CTCs isolation strategies have been developed such as size-based enrichment (e.g., Parsortix, ScreenCell^®^, and ISET^®^) and microfluidic-based enrichment (e.g., Parsortix, CTC-Chip/iChip, IsoFlux™, and GILUPI CellCollector™). The Parsortix system uses a combination of microfluidic and size/deformability-based approaches to separate CTCs from blood samples. Its main advantage over CellSearch^®^ is the ability to enumerate CTCs independently of their epithelial or mesenchymal status. However, cell size-based systems have difficulty to completely separate cancer cells and leukocyte by their size since patient’s CTCs exhibit a high degree of pleomorphism, where the size of the captured tumor cells can vary from 4-30 µm [20]. Therefore, size overlapping between CTCs and leukocytes will result in the loss of small CTCs. Besides, although CTCs can be detected in non-metastatic patients using both approaches, their low levels of non-advanced disease limit the application of these technologies as diagnostic tools [21].

In our cohort of patients with head and neck and NSCLC, we found that the Parsortix approach identified a higher proportion of CTC-positive patients than the CellSearch^®^ system, indicating that an antigen CTCs independent isolation approach can overcome the EpCAM expression heterogeneity that characterizes these tumors. Therefore, some non-epithelial or mesenchymal CTCs from cancer patients would be detected on the Parsortix platform but could escape the antigen-dependent enrichment method of the CellSearch^®^ EpCAM assay, and vice versa with epithelial CTCs, such as reflected our spiking experiments with different cell lines expressing different levels of EpCAM. Therefore, although the good results obtained using Parsortix system, a larger cohort of patients would be necessary to clearly determine which technology should be used to characterize CTCs from patients with both tumors.

Independently to the CTCs enumeration rate showed by both technologies, our study provides a proof-of-concept for the analysis of MET expression in CTCs to determine MET status in patients that might be eligible in clinical trial testing MET inhibitors, and could be useful to monitor the appearance of therapy resistance. Previously, Zhang et al. developed a noninvasive assay for MET+ CTCs capture and characterization from multiple patients with metastatic carcinomas using the CellSearch^®^ system [22]. Due to the strategy used, they identified a high prevalence of CD45-positive leukocytes expressing MET in patients with metastatic solid tumors, although the clinical relevance of this cell population is unknown. Ilie et al. also evaluated the prevalence of MET expression in CTCs from NSCLC patients [23], using a combined strategy similar to the one employed in the present work. In their study, MET expression was assessed using a CellSearch^®^ system and by immunocytochemistry on ISET-enriched CTCs from advanced-stage NSCLC patients. They found that MET expression was lower in CTCs analyzed by CellSearch^®^ than using ISET. In fact, only 9 of 256 (3.52%) patients showed CTCs with high MET expression (scores of 2^+^ or 3^+^) in CellSearch^®^, whereas MET-positive CTCs from advanced-stage NSCLC patients were successfully detected using the ISET platform, being 54 of the 75 (72%) patients’ blood samples defined as MET positive. Our data showed that the prevalence of MET expression in CTCs isolated by CellSearch^®^ is greater than previously reported data (20% MET-positive scores of 2+ or 3+), and even higher to the expected MET expression in patients with metastatic cancer. Importantly, using Parsortix system, we found 26.6% of patients with MET-positive scores of 2^+^ or 3^+^, being the concordance to detect the presence of CTCs overexpressing MET between both technologies, of 50%. These results showed that MET is expressed on CTCs with a considerable patient dependent and technology variability. Obviously, these results reflect the distinct CTCs enrichment methodologies of the two platforms, and the importance of tumor lineage for the MET-CTC-assay, highly diverse in our cohort of patient.

In addition to the CTCs analysis, we demonstrated the ddPCR approach to be a useful tool for detecting *MET* amplification in plasma samples from cancer patients. Zhang et al. had already shown this capacity, detecting *MET* CN in genomic DNA from cancer cell lines and tumor samples [24]. Recently, a study in cfDNA obtained from plasma of different tumor types showed that detection of MET alterations (amplification and point mutations) by liquid biopsy was feasible using the Guardant360 NGS panel [25]. In this study, they found 7% of patients with *MET* amplification in plasma, most of them with metastatic disease. Although NGS panels can provide more complete information regarding the status of other driver genes, our goal was the validation of a more sensitive and easier to implement test to monitor *MET* CN in plasma using ddPCR. For this aim, a cohort of healthy controls and cancer patients with different tumor types and metastatic stages were analyzed to detect *MET* CN by ddPCR. We found 16.4% of the metastatic patients and 3% of the non-metastatic setting as positive for *MET* amplification, being higher rates than the previously described using an NGS approach. Besides, we evaluated the concordance of the *MET* CN detection between plasma and tumor samples obtained at the same time as the disease evolution. Importantly, *MET* amplification positivity detected by ddPCR was comparable to that detected by FISH. Only one melanoma patient with *MET* amplification determined by FISH (MET/CEN-7 ratio = 6.1) did not show *MET* amplification in plasma using ddPCR (CN > 2.8). These results reinforced the value of the MET analysis in plasma samples as a more accessible monitoring tool to guide anti-MET therapies.

Actually, *MET* amplification is a relevant resistance mechanism to first-generation EGFR-TKIs, being detectable in approximately 5%-22% of NSCLC patients with acquired resistance to these drugs [26]. Osimertinib inhibits the EGFR variants Del19 and L858R, as well as the resistant T790M mutation. About 20% of such patients do not respond well to osimertinib, and almost all patients have eventually relapsed and developed resistance to the treatment. These resistance mechanisms are largely unknown, except for the C797S mutation and *MET* amplification [26]. There have been preclinical studies suggesting that monotherapy with osimertinib is not effective for the treatment of EGFR mutant NSCLC with acquired resistance to first-, second-, or third-generation EGFR-TKIs, due to *MET* amplification, or even MET hyperactivation [27]. Targeting EGFR and MET simultaneously may be required to overcome resistance to EGFR-TKIs by MET alteration, making it necessary to assess MET status before osimertinib treatment. Thus, some studies have reported the successful administration of dual EGFR and MET therapies [28,29,30]. The combination of osimertinib and MET inhibitors can be safe and effective in NSCLC patients with *MET* amplification detected by ddPCR as an acquired resistance mechanism. In this context, our results demonstrated the utility of plasma MET determination as a valuable biomarker for monitoring the appearance of resistance to anti-EGFR therapy. In fact, we reported a case of *MET* amplification detected in plasma from a NSCLC patient harboring EGFR L858R mutation after disease progression to erlotinib.

Regardless of the value of our findings, some limitations to our study should be kept in mind. The small number of tissue samples (obtained close to plasma collection) that were available did not allow us to carry out a robust comparative study between *MET* CN obtained from plasma and tissue. Despite this limitation, we showed that plasma may be a good option for monitoring the molecular evolution of the disease, which is even more relevant in patients for which a tissue-rebiopsy is not feasible. In addition, we implemented the detection of MET expression in CTCs from cancer patients using two complementary technologies and obtaining promising results. Nevertheless, taking into account the limited follow-up of the patients and the heterogeneity of MET overexpression found within the different tumor types and the technologies, the real clinical value of the assessment of MET expression in CTCs must be evaluated also in a larger cohort of patients.

In conclusion, we developed specific and noninvasive assays to monitor MET expression on CTCs and *MET* CN on cfDNA using blood from cancer patients. Both molecular alterations were detected in patients with different tumors and represent a good opportunity to characterize these tumors in a noninvasive and dynamic way, as well as guiding the addition of MET inhibitors.

## Figures and Tables

**Table 1:** Baseline demographics and clinical characteristics of the metastatic cancer patient population analyzed for *MET* amplification.

Features	All	Number of Lines of Treatment		Number of Metastatic Sites	
		≤1	>1	≤2	>2
**Gender**	N * (%) *MET* CN (Mean ± SD)	**N (%) *MET* CN (Mean ± SD)**	**N (%) *MET* CN (Mean ± SD)**	**N (%) *MET* CN (Mean ± SD)**	**N (%) *MET* CN (Mean ± SD)**
Male	83 (59.29%) 2.47 ± 0.40	48 (34.29%) 2.45 ± 0.36	31 (22.14%) 2.47 ± 0.46	36 (25.71%) 2.47 ± 0.45	44 (31.43%) 2.45 ± 0.34
Female	57 (40.71%) 2.59 ± 1.40	28 (20%) 2.42 ± 0.30	27 (19.29%) 2.81 ± 2.04	25 (17.86%) 2.35 ± 0.35	28 (20%) 2.87 ± 1.98
**Tumor type**					
NSCLC	77 (55%) 2.52 ± 1.21	34 (24.29%) 2.43 ± 0.29	36 (25.71%) 2.64 ± 1.75	28 (20%) 2.36 ± 0.31	42 (30%) 2.66 ± 1.61
Head and neck	30 (21.43%) 2.43 ± 0.37	28 (20%) 2.42 ± 0.38	1 (0.71%) 2.5	26 (16.43%) 2.40 ± 0.39	6 (4.29%) 2.52 ± 0.30
Colon	8 (5.71%) 2.65 ± 0.81	1 (0.71%) 2.52	6 (4.29%) 2.52 ± 0.87	2 (1.43%) 2.92 ± 1.65	5 (3.57%) 2.36 ± 0.38
Melanoma	6 (4.29%) 2.40 ± 0.49	4 (2.86%) 2.42 ± 0.50	2 (1.43%) 2.36 ± 0.68	3 (2.14%) 2.17 ± 0.12	3 (2.14%) 2.62 ± 0.66
Others **	19 (13.38%) 2.63 ± 0.	6 (4.29%) 2.63 ± 0.29	11 (7.86%) 2.68 ± 0.29	4 (2.86%) 2.85 ± 0.19	12 (8.57%) 2.60 ± 0.29

*N = 140 at baseline. ** Biliar (4), gastric (4), ovarian (3), renal (3), pancreas (3), retrocural (1), and skin (1) cancers. CN: copy number; SD: standard deviation; NSCLC: Non-small cell lung cancer.

## Supplementary Materials

The following are available online at https://www.mdpi.com/2073-4409/9/2/522/s1, Supplementary Figure S1: Study design for MET status assessment in metastatic patients; Supplementary Figure S2: Reproducibility of the *MET* CN determination by ddPCR; Supplementary Figure S3: Plasma *MET* CN values in the different metastatic cancers included in the study; Supplementary Figure S4: Correlation between EpCAM mRNA expression levels measured by Affymetrix microarrays and RNA-seq; Supplementary Figure S5: Immunofluorescence characterization of EpCAM in different tumor cell lines; Supplementary Table S1: Baseline demographics and clinical characteristics of the non-metastatic cancer patient’s population analyzed for *MET* amplification; Supplementary Table S2: Patient’s characteristics, CTCs detection, and MET expression on CTCs using CellSearch^®^ and Parsortix systems.
