# Supplementary material for: Detection of MET Alterations Using Cell Free DNA and Circulating Tumor Cells from Cancer Patients
Source: Cells. 2020 Feb 24;9(2):522. doi: 10.3390/cells9020522 (PMC7072825; doi:10.3390/cells9020522)
Supplement: Supplementary file 1 [file cells-09-00522-s001.pdf]

SUPPLEMENTAL MATERIAL

Supplementary Figure 1. Study design for MET status assessment in cancer patients.

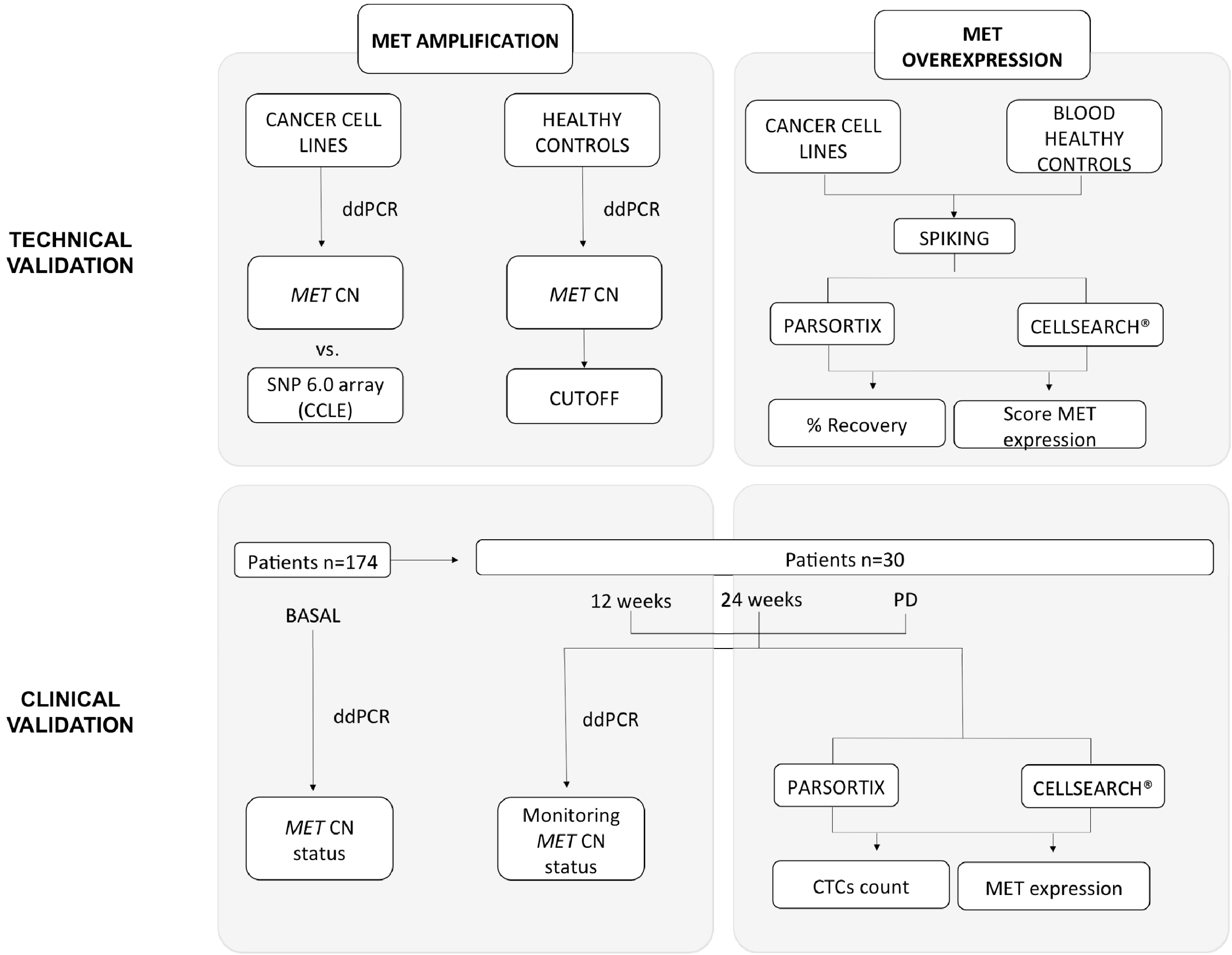

CN: copy number; CNV: copy number variation; ddPCR: digital droplet polymerase chain reaction; CCLE: cancer cell line encyclopedia; PD: progressive disease.

Supplementary Table 1. Baseline demographics and clinical characteristics of the non-metastatic cancer patient's population analyzed for *MET* amplification.

Feature		Feature	
**Gender**	**N*(%) *MET* CN (Mean±SD)**	**Tumor type**	**N*(%) *MET* CN (Mean±SD)**
Male	23 (67.65%)<br>2.40 ± 0.19	NSCLC	19 (55.88%)<br>2.34 ± 0.22
Female	10 (29.41%)<br>2.24 ± 0.27	Head and neck	4 (11.76%)<br>2.43 ± 0.18
		Colon	6 (17.65%)<br>2.31 ± 0.31
		Pancreas	4 (11.76%)<br>2.39 ± 0.0.22
		Melanoma	1 (2.94%)<br>2.46

*N=34 at baseline.

Supplementary Figure 2. Reproducibility of the *MET* copy number determination by ddPCR.

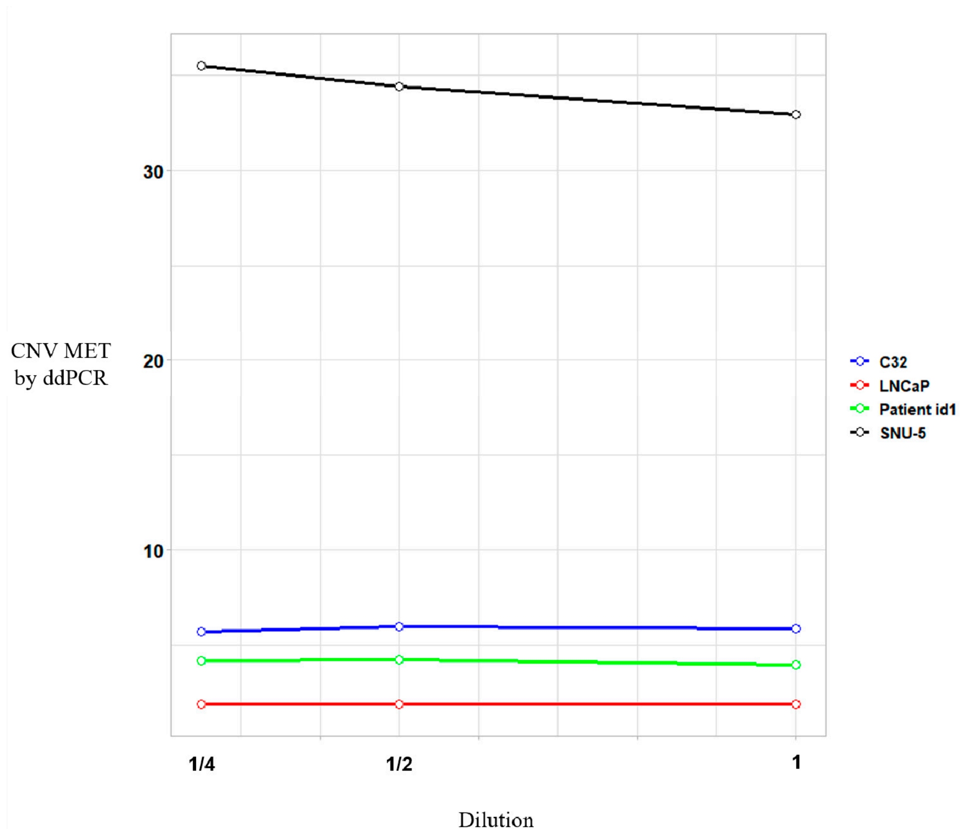

We represented some examples (3 cancer cell lines and a patient sample). *MET* CN values were determined by ddPCR in serial dilutions prepared by mixing the cancer cell lines (SNU-5, C32 and LNCaP) and the patient id1 sample with nucleic acid-free water. Circulating free DNA concentration of the patients id1 sample: dil_{1}=0.94 ng/ul; $dil_{1/2}$=0.47 ng/ul; $dil_{1/4}$=0.23 ng/ul. The minimum amount of DNA to detect *MET* amplification was 2.3 ng totals per each ddPCR reaction. Below of these values, there was no reproducibility of the results. All cfDNA samples used in the present study were above of this value in the ddPCR analysis.

Supplementary Figure 3. Plasma *MET* CN values in the different metastatic cancers included in the study.

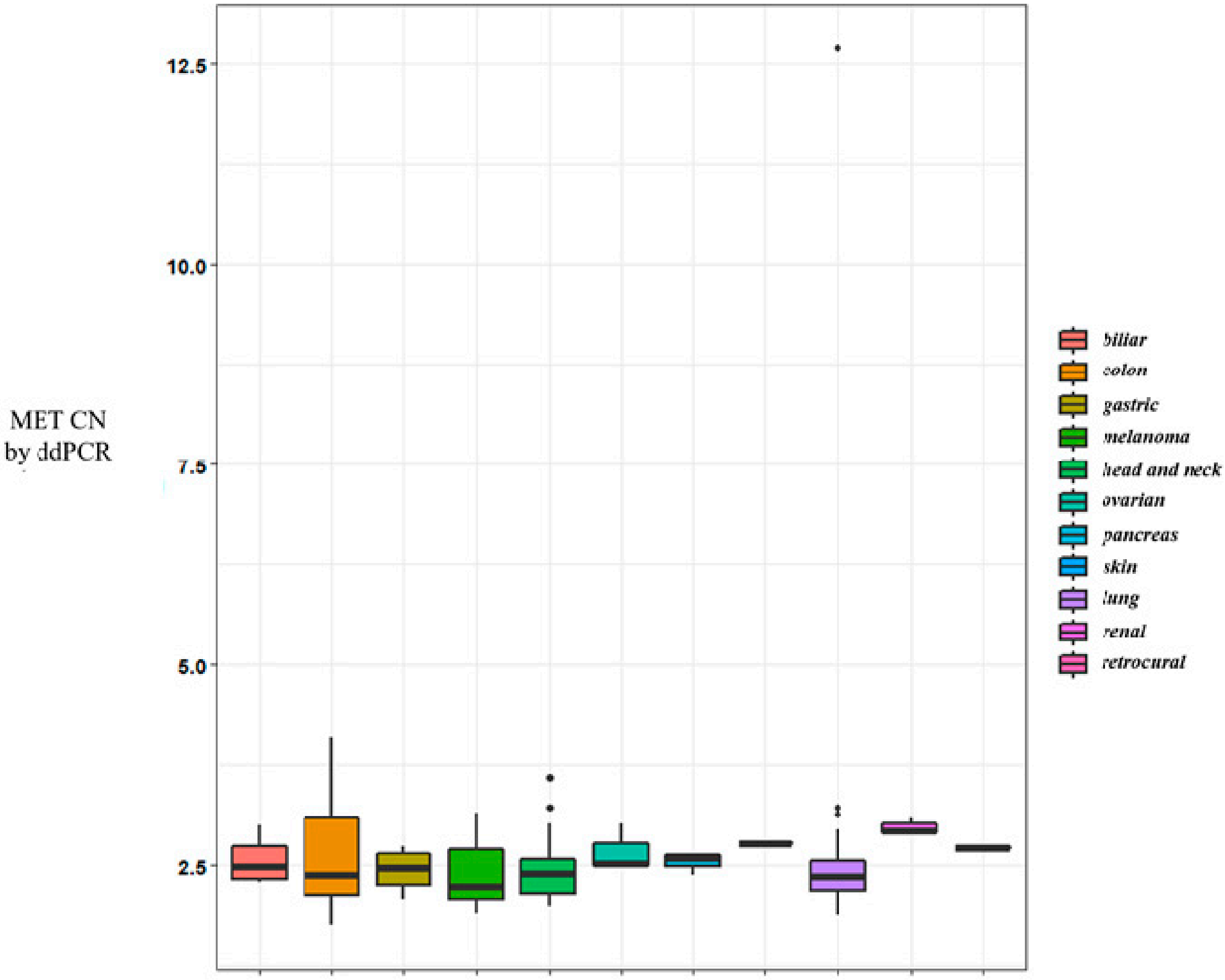

Supplementary Figure 4. Correlation between *EPCAM* mRNA expression levels measured by Affymetrix microarrays and RNA-seq.

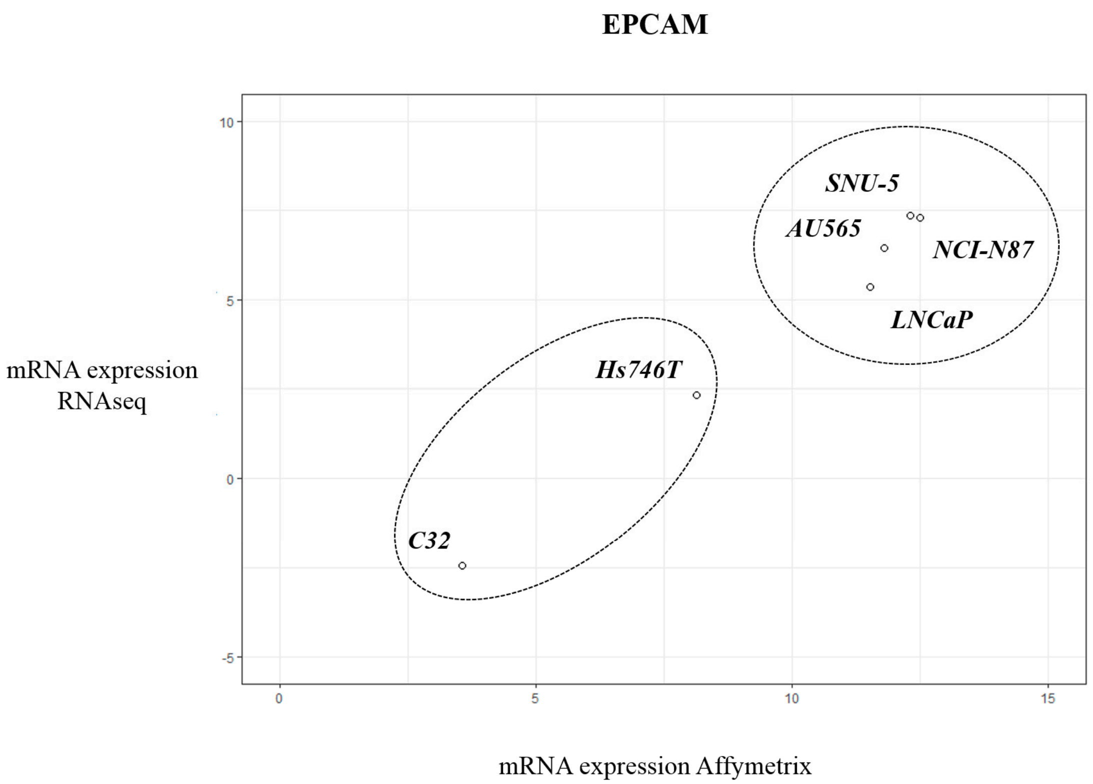

EpCAM expression data were extracted from the Cancer Cell Line Encyclopedia (CCLE) database (http://www.broadinstitute.org/ccle/home)

Supplementary Figure 5. Immunofluorescence characterization of EpCAM in cell lines.

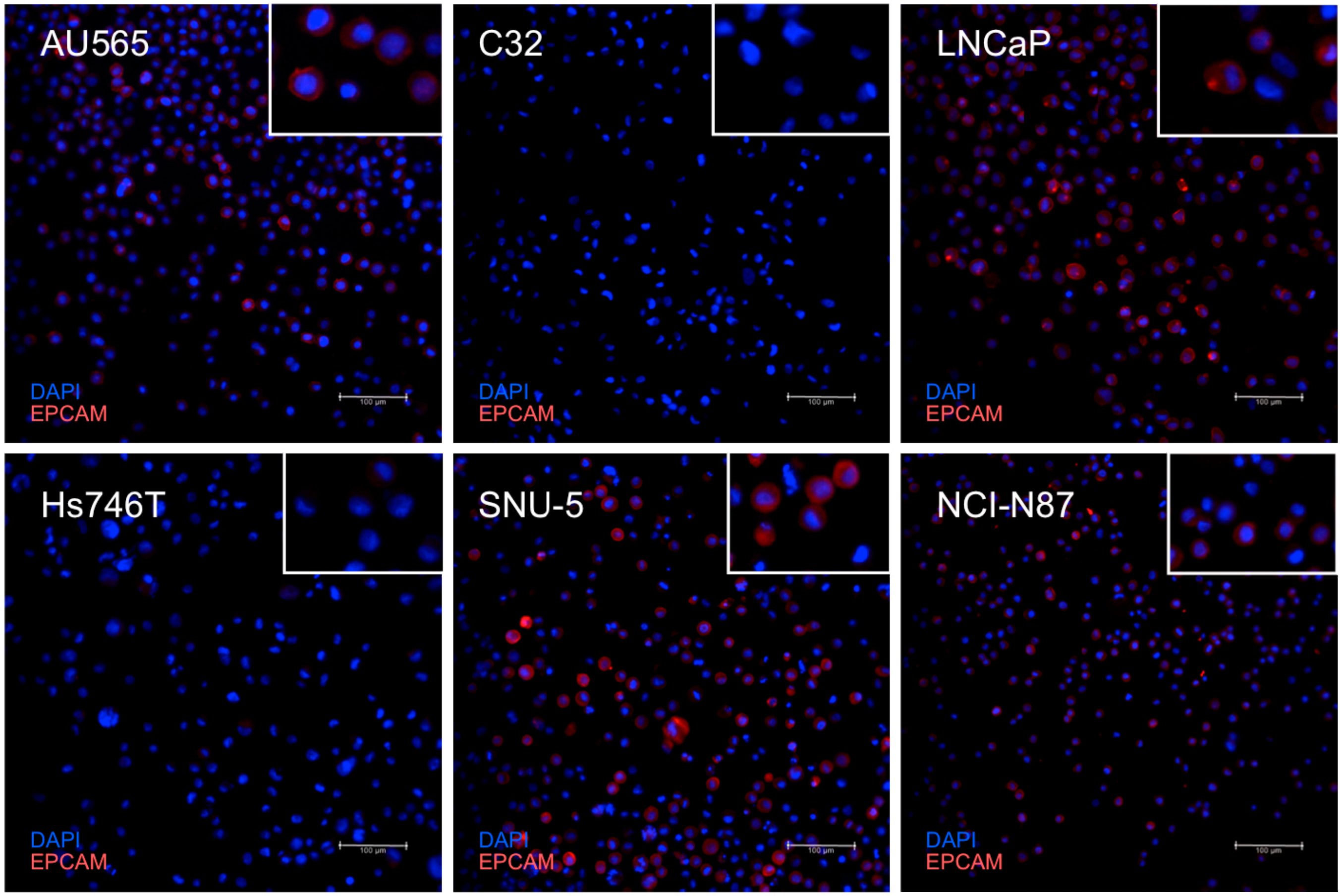

Supplementary Table 2. Tumor type, CTC detection and MET expression on CTCs using CellSearch® and Parsortix systems in patients with lung and head and neck tumors.

		CELLSEARCH®			PARSORTIX		
**Patient ID**	**Primary Tumor Location**	**CTC Total number**	**MET CTC Score**		**CTC Total number**	**MET CTC Score**	
			**0-1**	**2-3**		**0-1**	**2-3**
**5**	Lung	0	0	0	0	0	0
**6**	Head and neck	0	0	0	19	12	7
**9**	Lung	0	0	0	0	0	0
**11**	Lung	12	12	0	16	16	0
**14**	Lung	0	0	0	4	3	1
**18**	Lung	1	0	1	14	9	5
**22**	Head and neck	0	0	0	11	11	0
**26**	Lung	31	27	4	13	2	11
**28**	Lung	0	0	0	6	6	0
**31**	Head and neck	2	2	0	3	3	0
**37**	Head and neck	5	5	0	2	0	2
**42**	Head and neck	2	0	2	6	3	3
**48**	Head and neck	0	0	0	0	0	0
**51**	Head and neck	7	5	2	0	0	0
**52**	Head and neck	0	0	0	2	2	0
**53**	Head and neck	0	0	0	>50	>50	0
**56**	Head and neck	0	0	0	6	3	3
**61**	Head and neck	0	0	0	12	12	0
**64**	Head and neck	5	2	3	>50	>50	0
**67**	Lung	0	0	0	0	0	0
**70**	Head and neck	1	0	1	0	0	0
**71**	Head and neck	0	0	0	5	5	0
**72**	Lung	0	0	0	0	0	0
**75**	Lung	0	0	0	0	0	0
**80**	Lung	2	2	0	0	0	0
**81**	Lung	0	0	0	0	0	0
**82**	Lung	0	0	0	1	1	0
**90**	Lung	0	0	0	1	1	0
**91**	Lung	>50	NT	NT	6	0	6
**92**	Lung	0	0	0	1	1	0

NT: not tested.
